# Distinct Metabolic Endotype Mirroring Acute Respiratory Distress Syndrome (ARDS) Subphenotype and its Heterogeneous Biology

**DOI:** 10.1038/s41598-019-39017-4

**Published:** 2019-02-14

**Authors:** Akhila Viswan, Pralay Ghosh, Devendra Gupta, Afzal Azim, Neeraj Sinha

**Affiliations:** 1 Centre of Biomedical Research, SGPGIMS Campus, Raebarelly Road, Lucknow, 226014 India; 2 Faculty of Engineering and Technology, Dr. A. P. J. Abdul Kalam Technical University, Lucknow, 226021 India; 3 Department of Critical Care Medicine, Sanjay Gandhi Postgraduate Institute of Medical Sciences, Lucknow, 226014 India; 4 Department of Anaesthesia, Sanjay Gandhi Postgraduate Institute of Medical Sciences, Lucknow, 226014 India

## Abstract

Predisposing aetiologies in Acute Respiratory Distress Syndrome (ARDS), perpetuates to heterogeneous clinical course hampering therapeutic response. Therefore, physiological variables need to be identified by stratifying ARDS subphenotypes and endotype, to target ARDS heterogeneity. The present study is stimulated by the fact that the ARDS heterogeneity arises from diverse pathophysiological changes leading to distinct ARDS endotypes characterized by perturbed biological mechanism which can be exploited in terms of metabolic profile by metabolomics. Biological endotypes using (n = 464 patients and controls), mBALF and serum samples were identified by high – resolution NMR spectroscopy from two clinically diagnosed ARDS subtypes grouped under mild, moderate and severe ARDS as subphenotype1and pulmonary and extra – pulmonary ARDS as subphenotype2. The identified mBALF endotypes (isoleucine, leucine, valine, lysine/arginine, tyrosine, threonine) and serum endotypes (proline, glutamate, phenylalanine, valine) in both subphenotypes by statistical analysis were tested for their reproducibility and robustness. By combining metabolic endotypes with clinical based mortality score (APACHE and SOFA) added to their predictive performance as ARDS mortality predictors. Thus, a comprehensive set of mBALF endotypes representing compartmentalized lung milieu and serological endotypes representing systemic markers of ARDS subtypes were validated. The interlinked biological pathway of these disease specific endotype further elucidated their role as candidate biomarker in governing ARDS heterogeneous biology.

## Introduction

Spell of 50 years has gone since Acute Respiratory Distress Syndrome (ARDS) first description^1^ but the syndromes complexity and heterogeneity makes it the benchmark for intensivists at Intensive Care Unit (ICU)^2^. Advancement has been made in lung protective ventilation strategies like low tidal volume, prone positioning but effective pharmacotherapy is still suffering from backlog because of complex spectrum of diseases like sepsis, trauma, pancreatitis which all culminates into ARDS^3,4^. Bulk of ICU resources and healthcare are expended on ARDS patients on mechanical ventilation who occupy more than 25% of ICU with an overall mortality touching more than 40%^5,6^. Bedside care and cure becomes cardinal irrespective of cause because pathology is complex and heterogeneous. Clinical factors, observed radiologic appearances and pathology all ultimately affect the physiology thereby worsening or modifying outcome^7^. Therefore, personalized treatment necessitates first understanding the underlying physiology for better risk stratification. Failure of many clinical trials has prompted studies related to evidence based medicine and subsequently ARDS subphenotyping which is not met by clinical definition of ARDS^8^. Therapeutics maneuvour and tailored based therapies requires identifying measurable and distinct physiological differences in these ARDS subtypes^9–11^. ARDS subphenotypes has been grouped as mild, moderate and severe phenotype and pulmonary and extra-pulmonary phenotype based on underlying biology and severity^12^. These phenotypes have improved diagnosis but ARDS management, timely therapeutic intervention to portend progression and risk stratification requires distinct biological endotypes^13^. Differentiating the differential pathobiological mechanism characteristic of endotype is essential to reduce the inherent clinical and biological heterogeneity in ARDS^14^. The National Heart Lung and Blood Institute has also emphasized on the use of biological methodology and translational model to pave new directions in addressing ARDS heterogeneity and its resolution^15^. Therefore, need is to map biological endotype, which reflects physiological process to help stratify patient subsets as per ARDS severity/susceptibility. Endotype specific to the disease subtypes can be correlated to biomarker validation by epidemiological approaches.

In this respect, small molecular weight metabolites are the endpoint of all the biochemical and physiological process involved in metabolism. Their composition and variation in different biological matrices^16–20^ corresponding to drug, disease and toxic insult could reveal endotype of physiological relevance^21^. The miscellaneous etiology of ARDS incidence and acute diffuse onset necessitates dynamic and real time platform for timely and accurate clinical decision making under the framework of physiology besides conventional laboratory and radiological test^22^. Metabolomics provides an integrated view of metabolic fingerprint in a highly unbiased and reproducible manner^23^. Metabolomics provides the answer to the paradigm shift of identifying metabolic endotypes that best stratifies ARDS reflecting the underlying difference in biological mechanism due to pathophysiological stimuli, which ultimately results in different response to therapeutic intervention and therapies^24,25^. Dynamic changes and aberrations in response to the genetic, environmental and pathological stimuli are reflected in the metabolome which needs to be mapped by high throughput and high content analytical platform. Nuclear Magnetic Resonance (NMR) based metabolomics is a matter of choice in terms of it reproducibility, short analytical run and minimal sample preparation^26^. Thus we have employed NMR based metabolomics to identify metabolic endotypes, which can serve as biological marker mirroring the patient heterogeneity due to diverse pathophysiological process underlying ARDS subphenotype.

The aim of our present study was to identify endotype that best stratify ARDS phenotype with predictive power in determining favourable outcome and accurate and sensitive enough when associated with clinical variable. Therefore, in this study we have taken two clinically diagnosed subphenotypes of ARDS based on clinical manifestation and radiological appearances as mild, moderate and severe group categorized as subphenotype1 and pulmonary and extra-pulmonary group categorized as subphenotype2. To test the physiological difference in these two model n = 464 samples of mini Bronchoalveolar lavage fluid (mBALF) and serum biofluid were collected and subjected to metabolic profiling by NMR. Though both these biofluids vary in the mode of collection and biomarker information but together depict comprehensive view of ARDS biology by providing both circulating biomarkers of systemic origin and localized biomarkers of lung milieu. The best identified mBALF and serum endotypes of different subphenotypes were tested for their reproducibility and robustness in test set and training set correlated to each subphenotype. The characterized endotypes were substantiated in predicting outcome by their classification in survivor and non-survivor group. Their clinical relevance were verified by combining with mortality score in area under the receiver operating characteristic curve (AUROC). The underlying complex interplay of biological process was examined in model based on metabolic endotype of ARDS using integrated metabolic pathway. Thus metabolomics proves to be platform in mapping ARDS heterogeneous biology by determining the predictive endotype.

## Results

### Patient characteristics

**Figure 1:**
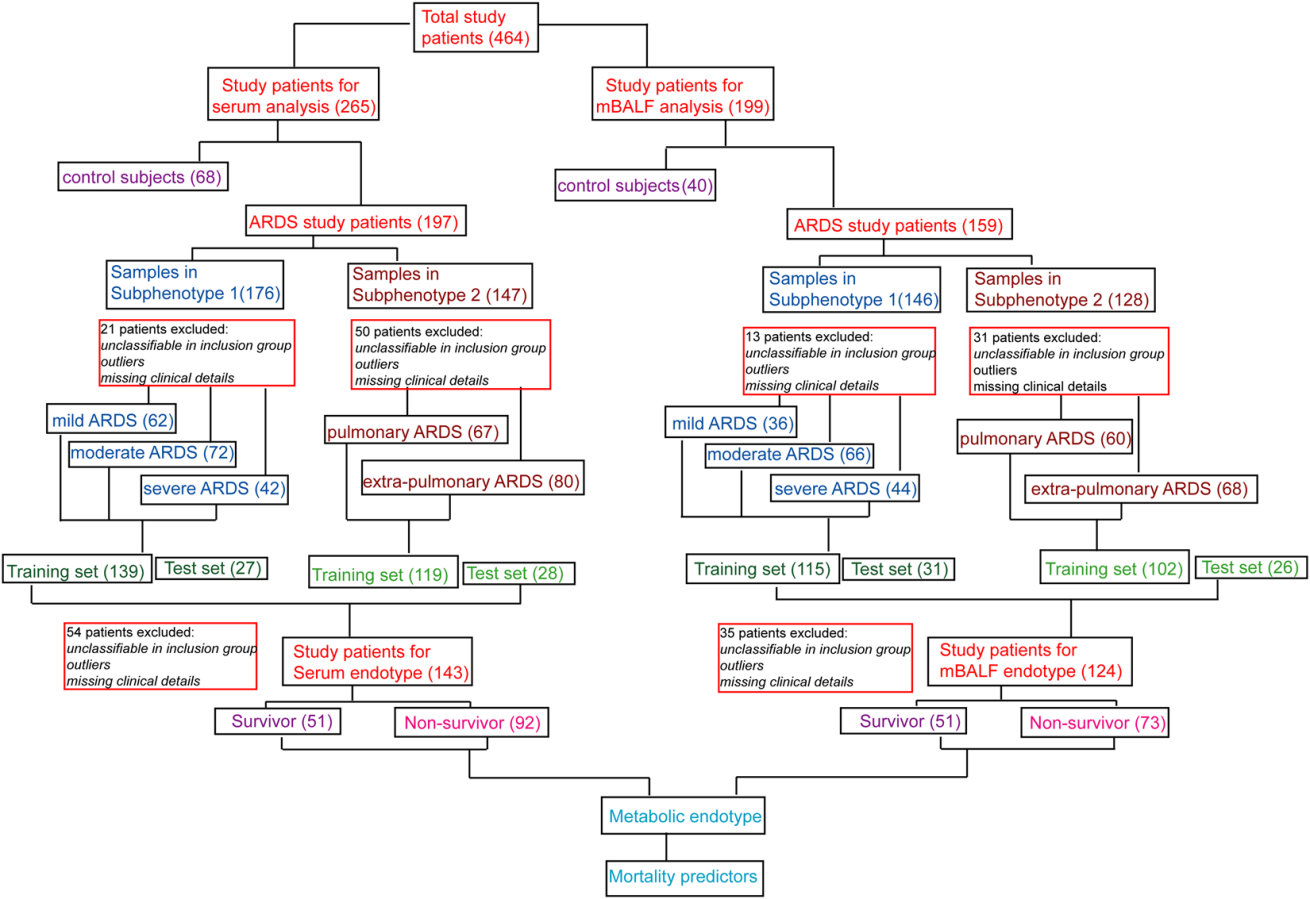
Flow chart depicting the sample size of serum and mBALF of control and ARDS patients enrolled in the present study.

The patients baseline characteristics and clinical variables stratified for study population at the time of sampling are provided in Table 1 which includes age matched demographic characteristics, Acute Physiology and Chronic Health Evaluation-II (APACHE II) and Sequential Organ Failure Assessment (SOFA) score. The mild and moderate and severe ARDS patients were grouped and diagnosed as per the new convened Berlin definition. Pulmonary and extra pulmonary subphenotype grouping was governed by clinical symptoms and its ARDS association. The patient included in the study group is shown through the flow chart (Fig. 1) depicting the included size of patients and some excluded subjects because of missing and confounding clinical details.

**Table 1:** Clinical and baseline characteristics of subjects.

Clinical characteristics	Survivor (51)	Non-survivor (92)	
**Outcome study patients for serum endotype in ARDS (n = 143)**			
Age	43.11 ± 1.76	46.9 ± 1.68	
Sex (M/F)	31/20	59/33	
Primary diagnosis, (n)	Neurological (10)	Neurological (9)	
	Respiratory (16)	Respiratory (34)	
	Cardiac (5)	Cardiac (5)	
	Gastrointestinal (15)	Gastrointestinal (30)	
	Tropical infections (4)	Tropical infections (10)	
	Others (1)	Others (4)	
Comorbidities, (n)	Diabetes mellitus (12)	Diabetes mellitus (18)	
	Hypertension (9)	Hypertension (12)	
	Diabetes + hypertension (14)	Diabetes + hypertension (28)	
	Hypothyroidism (4)	Hypothyroidism (9)	
	Chronic kidney disease (3)	Chronic kidneydisease (5)	
	Chronic artery disease (2)	Chronic artery disease (3)	
	Nil (7)	Nil (17)	
APACHE-II*	13.56 ± 0.28	17.64 ± 0.29	
SOFA*	7.78 ± 0.22	10.25 ± 0.20	
TLC*	11.70 ± 0.73	19.94 ± 1.23	
PCT*	1.87 ± 0.28	3.70 ± 0.65	
Serum creatinine*	2.04 ± 0.23	2.80 ± 0.14	
Bilirubin	1.40 ± 0.13	1.62 ± 0.09	
Length of hospital (days) stay	21.94 ± 1.04	23.53 ± 1.11	
Days of mechanical ventilation	15 ± 1.12	18.14 ± 1.01	
Length of ICU stay (days)	17.92 ± 1.09	19.10 ± 1.04	
**Total study patients for serum analysis (n** **= 265)**			
	**Control subjects (n = 68)**	**ARDS patients (n = 197)**	
Age	48.33 ± 1.91	48.87 ± 1.14	
Sex	42/26	111/86	
**ARDS Subphenotype for serum samples**			
**Subphenotype 1 (n = 176)**	**Mild (62)**	**Moderate (72)**	**Severe (42)**
P/F ratio*	248.29 ± 4.1	165.22 ± 3.2	84.14 ± 2.2
Age	44.61 ± 2.1	47.125 ± 1.9	49.30 ± 2.2
Sex (M/F)	37/25	44/28	27/15
**Subphenotype 2 (n = 147)**	**Pulmonary etiologies (67)**		**Extra-pulmonary etiologies (80)**
Age	46.89 ± 1.91		42.63 ± 1.80
Sex (M/F)	43/24		48/32
**Outcome study patients for mBALF endotype in ARDS (n = 124)**			
**Clinical characteristics**	**Survivor (51)**	**Non-survivor (73)**	
Age	43.09 ± 1.81	47.78 ± 2.01	
Sex (M/F)	36/15	42/31	
Primary diagnosis, (n)	Neurological (8)	Neurological (6)	
	Respiratory (20)	Respiratory (30)	
	Cardiac (3)	Cardiac (1)	
	Gastrointestinal (13)	Gastrointestinal (24)	
	Tropical infections (5)	Tropical infections (9)	
	Others (2)	Others (3)	
Comorbidities, (n)	Diabetes mellitus (10)	Diabetes mellitus (16)	
	Hypertension (6)	Hypertension (10)	
	Diabetes + hypertension (14)	Diabetes + hypertension (21)	
	Hypothyroidism (5)	Hypothyroidism (7)	
	Chronic kidneydisease (3)	Chronic kidneydisease (4)	
	Chronic artery disease (3)	Chronic artery disease (1)	
	Nil (10)	Nil (14)	
APACHE*	13.96 ± 0.28	17.50 ± 0.32	
SOFA*	7.09 ± 0.22	10.08 ± 0.23	
TLC*	12.18 ± 0.83	20.86 ± 1.45	
PCT*	1.59 ± 0.21	3.97 ± 0.82	
Serum creatinine*	2.03 ± 0.23	2.83 ± 0.18	
Bilirubin	1.37 ± 0.11	1.64 ± 0.10	
Length of hospital (days) stay	24.8 ± 1.20	25.24 ± 1.13	
Days of mechanical ventilation	16.92 ± 1.08	18.52 ± 1.09	
Length of ICU stay (days)	20.27 ± 1.23	19.09 ± 1.10	
**Total study patients for mBALF analysis (n = 199)**			
Study patients	Control (n = 40)	ARDS (n = 159)	
Age	47.8 ± 2.27	46.03 ± 1.24	
Sex	25/15	89/70	
**ARDS Subphenotype for mBALF samples**			
**Subphenotype 1 (n = 146)**	**Mlild (36)**	**Moderate (66)**	**Severe (44)**
P/F ratio*	247.91 ± 5.6	159.18 ± 3.7	80.31 ± 2.9
Age	46.22 ± 2.8	44.9 ± 2.0	49.15 ± 2.1
Sex (M/F)	22/14	41/25	25/19
**Subphenotype 2 (n = 128)**	**Pulmonary etiologies (60)**		**Extra-pulmonary etiologies (68)**
Age	43.28 ± 2.0		47.55 ± 1.90
Sex (M/F)	38/22		45/23

Data presented as mean + standard error.

P/F Ratio: Partial pressure of oxygen to the fraction of inspired oxygen ratio.

APACHE-II: Acute Physiology and Chronic Health Evaluation-II.

SOFA: Sequential Organ Failure Assessment.

*Indicates variables which statistically significant.

### ARDS serum and mBALF predictability

**Figure 2:**
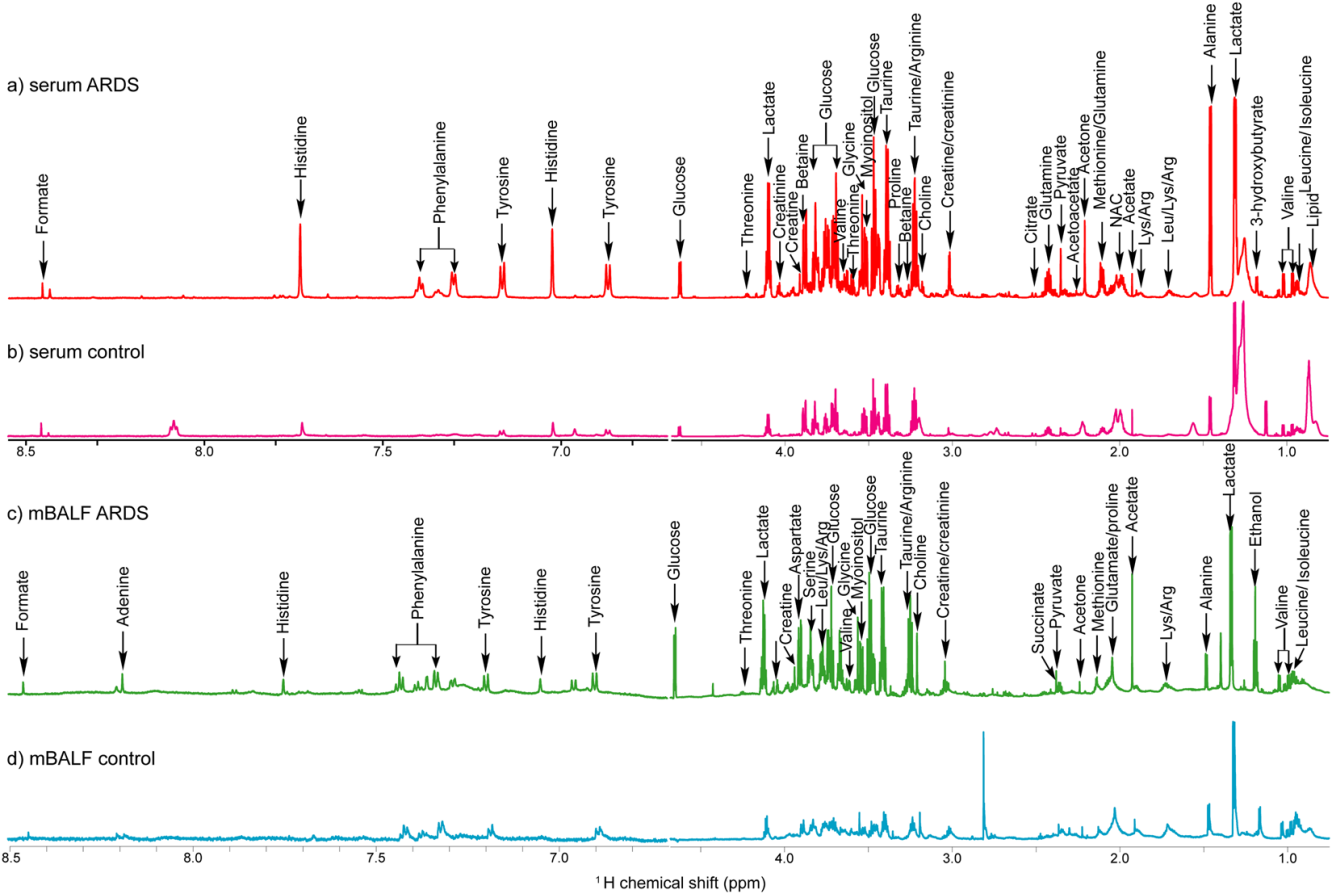
Representative ^1^H NMR spectrum of (**a**) serum ARDS patient, (**b**) serum control, (**c**) mBALF ARDS patient and (**d**) mBALF control.

The NMR spectroscopy was performed on all the collected samples of both mBALF (n = 199) and serum (n = 265) for the identification and assignment of metabolites present in ARDS with respect to control. A representative NMR spectra of serum control and serum ARDS sample is shown along with mBALF control and mBALF ARDS (Fig. 2). The difference in metabolite intensity indicates difference in ARDS associated changes with respect to controls, which can be mapped by chemmometric analysis to further validate the perturbed metabolism. We initially tested the accuracy of serum as diagnostic fluid from n = 265 subjects in discriminating n = 68 control and n = 197 ARDS patients. As a result, we obtained clear separation in OPLS-DA with R2Y = 0.80 and Q2 = 0.76 and PLS-DA as shown in supplementary information (Figs SI 1c,d, 3a–e). Similarly, mBALF as a diagnostic fluid was established for n = 199 subjects in discriminating n = 40 control and n = 159 ARDS patients with R2Y = 0.92 and Q2 = 0.76 value by OPLS-DA and PLS-DA (Figs SI 1a,b and 2a–e). Small value of Q2 can be attributed to small size of control mBALF samples. The resulting separation prompted towards the first objective of our study to stratify ARDS in two subphenotypes based on clinical characteristics and its predictability in determining metabolic endotype using NMR based metabolomics regardless of clinical heterogeneity.

### ARDS subphenotypes

Firstly, hypoxemic partial pressure of oxygen to the fraction of inspired oxygen (P/F) ratio was used to build subphenotype1 in both mBALF and serum categorizing patients on the basis of severity involving mild, moderate and severe subjects with P/F ratio between 200–300, P/F ratio between 100–200 and P/F ratio less than 100 respectively. Secondly, different respiratory mechanism of lung injury, was used as a basis to build subphenotype2 in both mBALF and serum involving pulmonary aetiologies (pneumonia, lung contusion) called direct ARDS and extra-pulmonary (pancreatitis, trauma, sepsis) contributors known as indirect ARDS.

### Subphenotype1 metabolic profile

**Figure 3:**
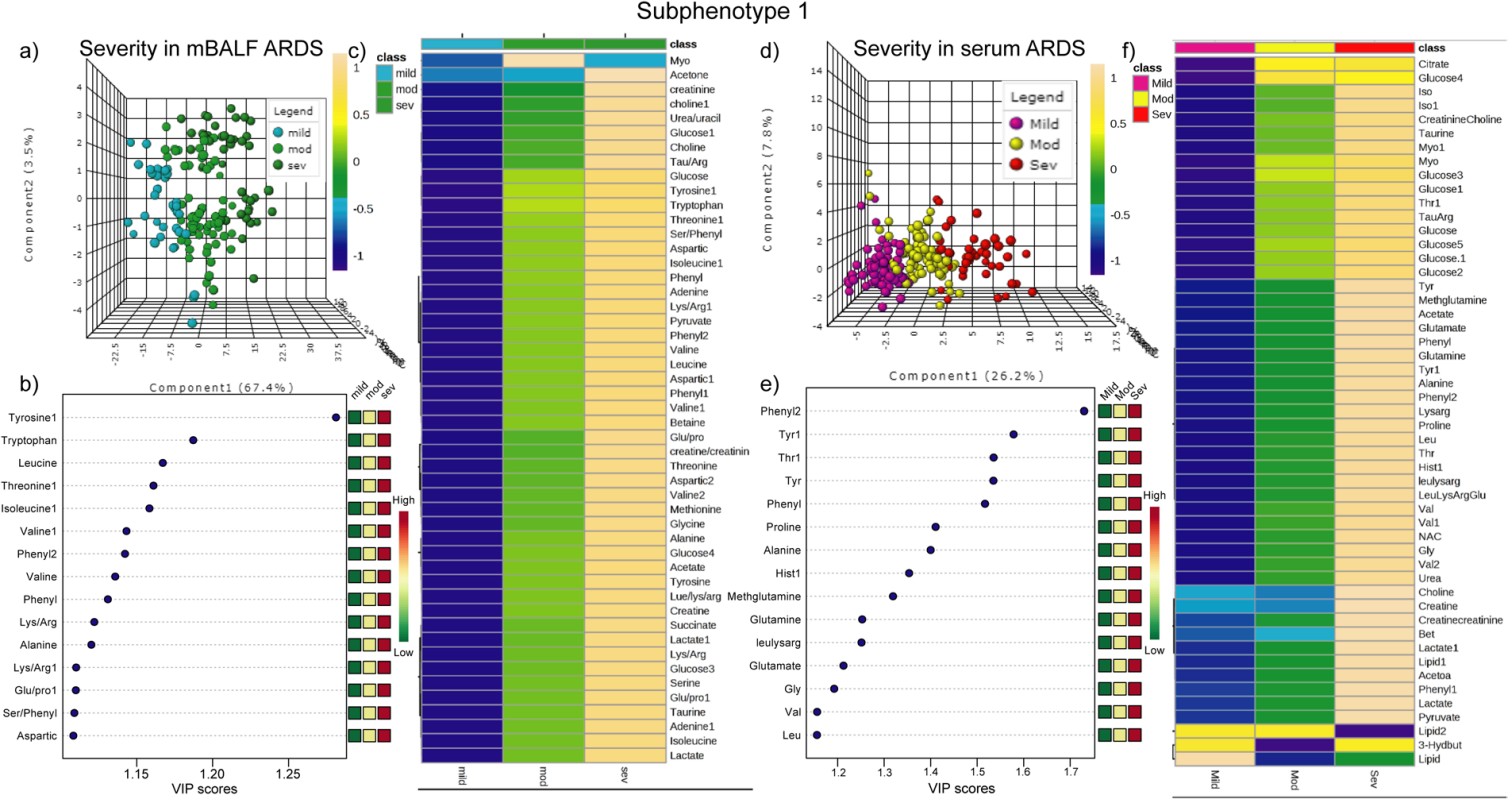
Subphenotype 1 of ARDS based on P/F ratio shown as (**a**) 3D score plot of PLS-DA (**b**) VIP plot and (**c**) heat map in mBALF and (**d**) 3D score plot of PLS-DA (**e**)VIP plot and (**f**) heat map in serum.

In subphenotype1, n = 176 serum specimen grouped in to n = 62 mild ARDS, n = 72 moderate ARDS and n = 42 severe ARDS patients were subjected to epidemiological studies whereas in n = 146 mBALF specimens, n = 36 mild ARDS, n = 66 moderate ARDS and n = 44 severe ARDS patients were grouped for analysis Fig. 3a,d. R2 = 0.77 and Q2 = 0.71 in mBALF and R2 = 0.80 and Q2 = 0.74 in serum along with permutation test statistics and classification error rate (Fig. SI 6a–h) were obtained as a reliable index to deduce the accuracy of the above model. After subsequent one-way ANOVA based univariate analysis with *p* value less than 0.05 as threshold and VIP plot based on value more than 1 were used as a final measure to determine the metabolites responsible to differentiate the different subtypes, as shown in Fig. 3b,e. Heat map (Fig. 3c,f) using person correlation and wards linkage shows the visual interpretation of change in metabolite intensity as the disease progress from mild to moderate and severe ARDS. Thus, NMR based metabolomics proved to be a pivotal platform in validating the above clinical model in terms of metabolic profile. Separation observed in subphenotype 1 with different degree of accuracy indicates the utility of both biofluids in reflecting the underlying physiology.

### Subphenotype2 metabolic profile

**Figure 4:**
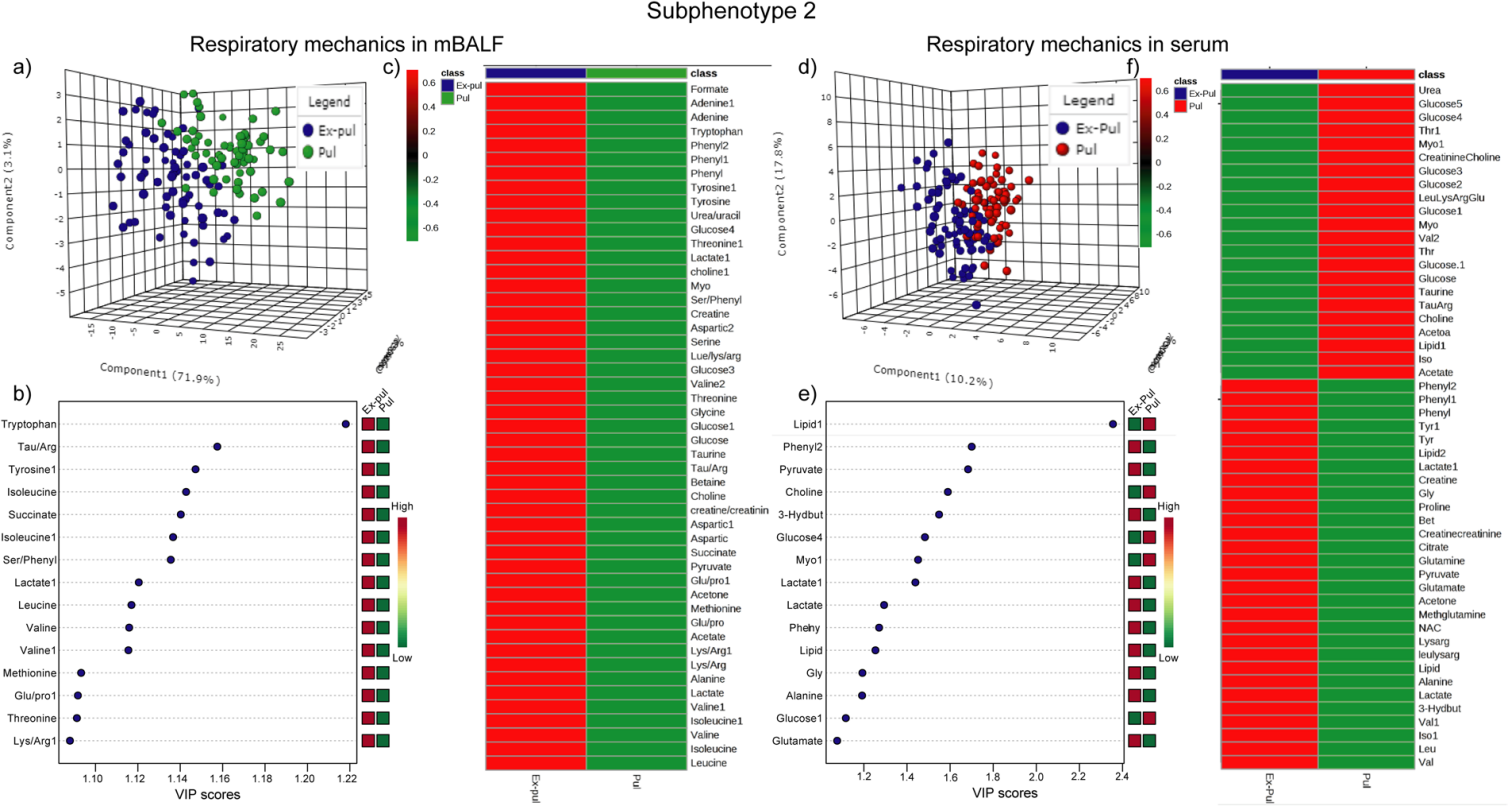
Subphenotype 2 of ARDS based on direct and indirect ARDS due to pulmonary and extra-pulmonary etiology shown as (**a**) 3D score plot of PLS-DA (**b**) VIP plot and (**c**) heat map in mBALF and (**d**) 3D score plot of PLS-DA (**e**) VIP plot and (**f**) heat map in serum.

In subphenotype2, n = 128 mBALF specimens were sorted in n = 60 pulmonary etiology and n = 68 extra-pulmonary etiology of ARDS whereas n = 147 ARDS serum specimens were classified into n = 67 pulmonary origins and n = 80 extra – pulmonary origins for subsequent analysis. Accuracy of 0.86, R2 = 0.63 and Q2 = 0.51 in mBALF and accuracy of 0.81, R2 = 0.60 and Q2 = 0.48 in serum along with permutation test statistics and classification error rate (Fig. SI 7a–h), proves to be a significant threshold for considering the reliability of model of ARDS based on different etiology (Fig. 4a,d). Heat map plot shows the difference in metabolic intensity in mBALF compared to serum in terms of information pertaining to diffused ARDS lungs from pulmonary and extra – pulmonary causes (Fig. 4c,f). This is due the fact that mBALF represent mostly unambiguous lung related perturbations as taken from proximal alveoli. Whereas serum is more generalized and broad in drawing useful information correlated to spread of ARDS specific inflammation, infection or pathogenesis. So both the fluid should be evaluated for wider choice and comprehensive set of markers. The above-mentioned subphenotype2 highlights the fact that the different origin or etiology affect the final underlying lung injury in ARDS. After affirming its significance from t-test and on the basis of VIP plot with value more than 1(Fig. 4b,e), significant metabolites were grouped.

### Validation of subphenotype1 and subphenotype2

To validate the accuracy of subphenotype1 and subphenotype2, the cohort was randomly divided into training set and test set following 80/20 holdout ratio^27^. mBALF severity model comprised n = 115 in training set and n = 31 in test set with 0.89 and 0.70 accuracy respectively. (Fig. SI 4a–d). Meanwhile mBALF based respiratory model comprised n = 102 in training set and n = 26 in test set with 0.82 and 0.56 accuracy respectively (Fig. SI 4e–h). Serum based severity model in subphenotype 1 comprised n = 139 in training set and n = 27 in test set with 0.78 and 0.67 accuracy respectively (Fig. SI 5a–d). Meanwhile serum depicting respiratory mechanics included n = 119 in training set and n = 28 in test set for subphenotype2 with 0.83 and 0.90 accuracy respectively (Fig. SI 5e–h). The above analysis concluded significant metabolites same as obtained in the validation cohort establishing the reproducibility of the endotypes obtained from mBALF and serum.

Consequently, the serum specific and mBALF specific metabolic profile obtained and its validation in test set and training set highlighted biological and physiological heterogeneity in ARDS which resulted in distinct subset of metabolites in both subphenotypes corresponding to different biofluids as shown in Table 2. All the metabolites were selected and considered statistically significant after log transformation and Pareto scaling based on p value less than 0.05 and VIP value more than 1 with their mean values and standard deviation reported before normalization in SI Tables 1–4.

**Table 2:** List of significant metabolites from subphenotype 1 and subphenotype2 in mBALF and serum.

mBALF Metabolites		Serum Metabolites	
*Subphenotype1*	*Subphenotype2*	*Subphenotype1*	*Subphenotype2*
Lysine/arginine	Isoleucine	Tyrosine	3-hydroxybutyrate
Alanine	Leucine	Phenylalanine	Lactate
Isoleucine	Valine	Leucine/Lysine/Arginine	Alanine
Leucine	Lactate	Methglutamine	Lipid
Phenylalanine	Lysine/Arginine	Alanine	Glutamate
Valine	Methionine	Glutamine	Pyruvate
Tyrosine	Pyruvate	Valine	Choline
Glutamate/proline	Succinate	Proline	Glucose
Aspartic	Betaine	Histidine	Glycine
Tryptophan	Taurine/Arginine	Leucine	Myoinositol
Threonine	Threonine	Glycine	Phenylalanine
Serine/Phenyalnanine	Serine/Phenyalanine	Glutamate	Valine
Lactate	Myoinositol	Threonine	Proline
Methionine	Tyrosine	Isoleucine	Leucine
	Tryptophan		Isoleucine
	Glutamate/proline		Threonine

### mBALF and serum endotype as mortality predictors

**Figure 5:**
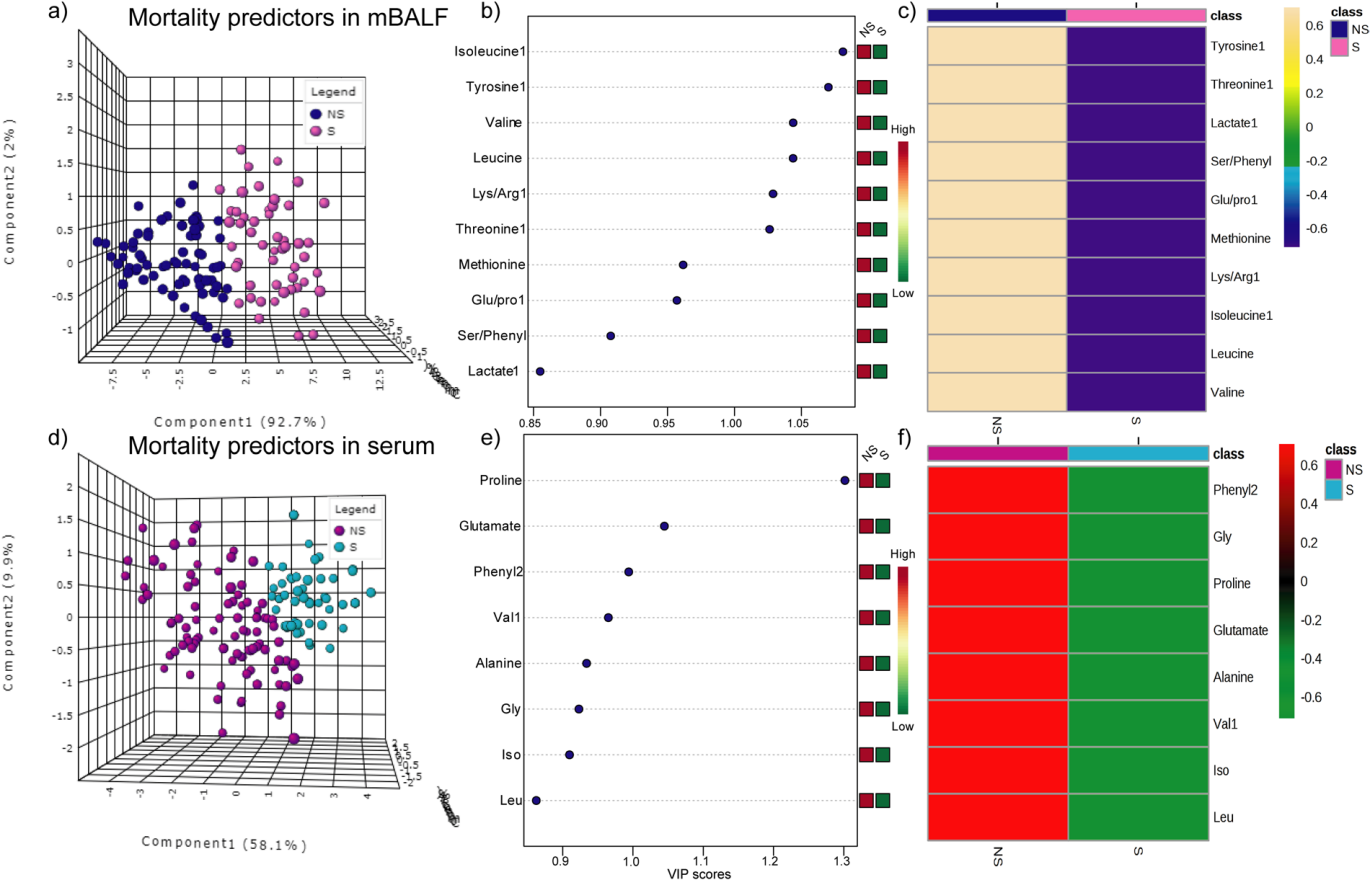
mBALF endotype shown as (**a**) 3D score plot of PLS-DA (**b**) VIP plot and (**c**) heat map and serum endotype (**a**) 3D score plot of PLS-DA (**b**) VIP plot and (**c**) heat map in classification of mortality subgroup.

Significant metabolites common to mBALF subphenotype 1 and subphenotype 2 representing lung specific disease pathophysiology were grouped as mBALF endotype. Similarly, metabolites common to subphenotype1 and subphenotype2 in serum representing ARDS directed serological changes were grouped as serum endotype. Endotypes are representative of distinct functional and pathobiological mechanism but how well they respond to outcome needs to be ascertained to determine their clinical applicability. Therefore, we designed the predictive model based on survivor and non-survivor groups in ARDS for both in mBALF and serum biofluids. The analysis was performed to discover the final set of markers representing ARDS mortality. The mBALF endotype, isoleucine, tyrosine, valine, leucine, lysine/arginine, threonine, glutamate/proline, serine/phenylalanine and lactate were used in the predictive model of survivor and non-survivors (Fig. 5a). Similarly, serum endotype, proline, glutamate, phenylalanine, valine, alanine, glycine, isoleucine and leucine were used to determine their role in differentiating survivor and non-survivor patients (Fig. 5d). mBALF taken from n = 124 patients included n = 51 survivors and n = 73 non survivors whereas n = 143 samples of serum included n = 51 survivors and n = 92 non-survivors ARDS subjects. Further t-test and subsequent PLS-DA analysis sorted out panel of metabolic endotype representative of ARDS heterogenous biology related to mortality (Fig. 5b,e). mBALF endotype based model resulted in R2 = 0.72, Q2 = 0.69 and accuracy 0.98 whereas serum endotype based resulted in R2 = 0.67, Q2 = 0.63 and accuracy 0.95 along with permutation test statistics and classification error rate shown in Fig. SI 8a–h. Pearson correlation based heat map showed amplified metabolite intensity in the non-survivor subjects compared to survivors implying greater metabolic dysfunction attributed by mBALF and serum endotypes as seen in non-survivors (Fig. 5c,f).

### Accuracy of ARDS metabolic endotype

**Figure 6:**
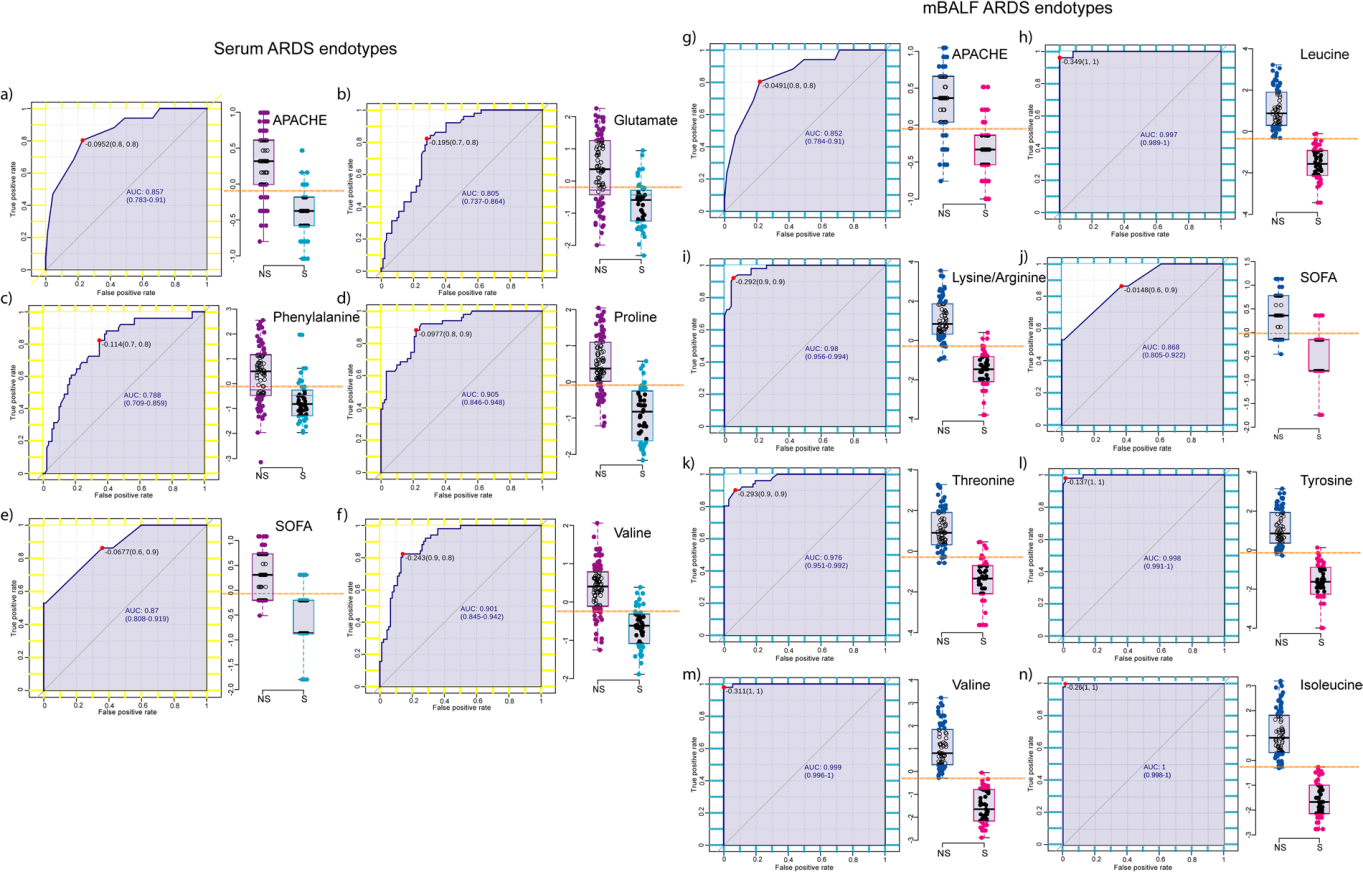
Box whisker plot and ROC curve of individual (**a–f**) serum endotype and (**g–n**) mBALF endotype along with APACHE and SOFA score.

**Figure 7:**
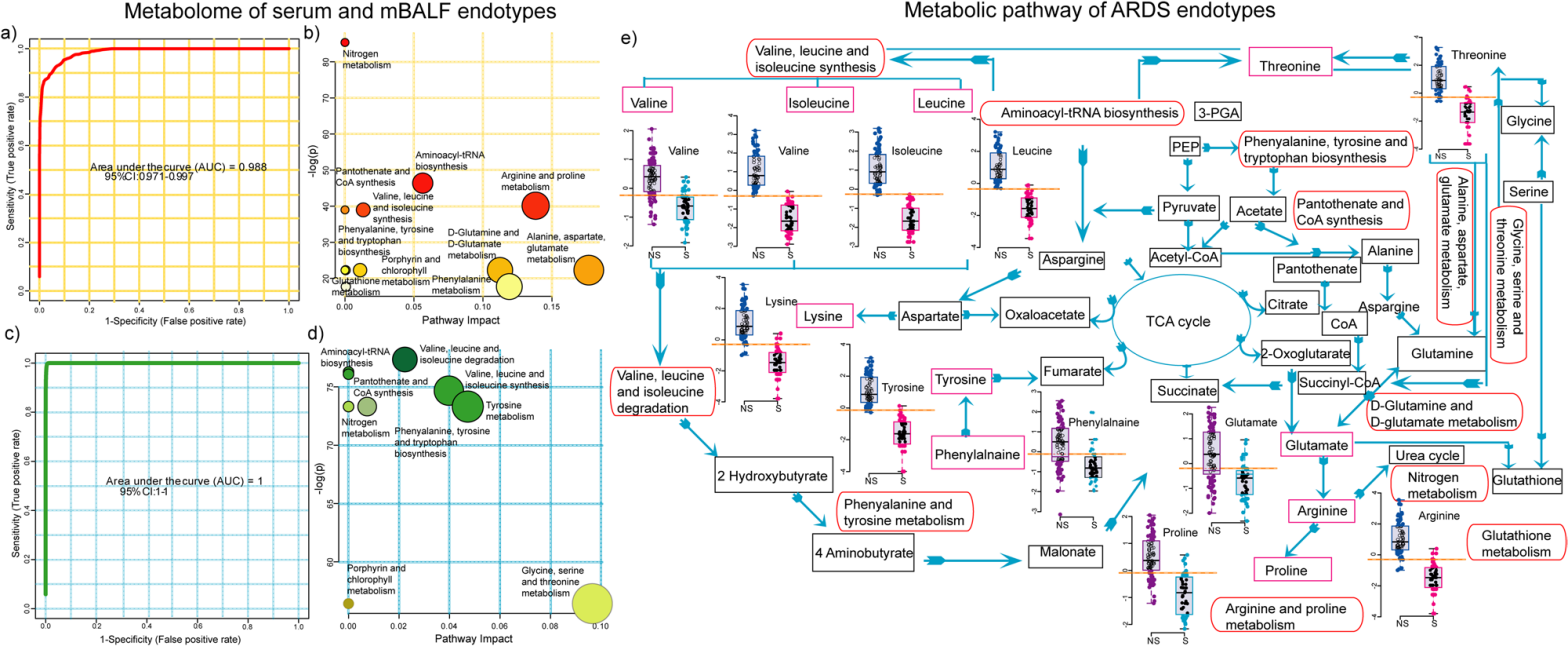
(**a**,**c**) AUROC of cumulative serum endotype and mBALF endotype with clinical score (**b**,**d**) over represented metabolic pathway of ARDS associated changes in serum and mBALF (**e**) integrated biological network of ARDS.

Box whisker plot and ROC of individual metabolic endotypes including proline, glutamate, phenylalanine, valine of serum and isoleucine, tyrosine, valine, leucine, lysine/arginine and threonine of mBALF were plotted to validate their relative concentration with respect to outcome group along with clinical score APACHE and SOFA respectively as shown in Fig. 6(a–n). Box whisker plot showed relative high metabolite concentration in non-survivor subjects highlighting increase in metabolite aberration in patients susceptible to mortality. Accuracy was further substantiated by individual ROC values specifying their role as mortality predictors. The above sensitivity and specificity of individual serum metabolites and mBALF metabolites as resultant serum and mBALF endotype were used to determine their clinical predictability when combined with clinical based APACHE and SOFA score. The accuracy increased to AUROC 1 indicating the clinical relevance of the above determined metabolic endotype (Fig. 7a,c).

### ARDS metabolic network

Pathway analysis of serum endotype and mBALF endotype predictive of mortality gave important metabolic pathway symbolic of ARDS correlated changes in metabolism. Figure 7b illustrates most symptomatic pathways of circulatory serum based endotype model characteristic of systemic changes as a consequence ARDS infection, inflammation and pathogenesis. Similarly, Fig. 7d reflects most implicated pathways of localized mBALF based endotype model representative of ARDS diffused lung compartment due to pulmonary edema and infiltration of neutrophils and other inflammatory mediators. Nitrogen metabolism, aminoacyl tRNA synthesis, valine, leucine and isoleucine synthesis, pantothenate and CoA synthesis, phenylalanine, tyrosine and tryptophan biosynthesis common to mBALF and serum endotype indicated anomalous pathobiological mechanism due to ARDS (Fig. 7b,d). Thus all the cumulative metabolic endotypes representative of ARDS pathophysiological changes and outcome were used to build an integrated metabolic pathway showing the conclusive metabolites and their contribution in governing ARDS heterogeneous biology (Fig. 7e). As a result, we obtained distinct class of metabolic endotypes identified by metabolomics using mBALF and serum that can clearly distinguish ARDS subtypes with its strong correlation to mortality.

## Discussion

ARDS is a complex syndrome where pathophysiology is interwoven with pathology and underlying lung injury is common regardless of the type of multiple insults. In case of ARDS, the critical illness leads to severe derangement of homeostasis and haemodynamic equilibrium ultimately affecting the downstream metabolic process and interlinked pathways. Metabolic dysregulation is the outcome of upstream physiological and pathophysiological changes affecting whole system corresponding to endogenous or exogenous insult. Different degrees of anatomical and physiological derangement add to clinical and biological heterogeneity which requires characterization of disease specific endotype by epidemiological approaches. Both mBALF and serum has been previously established as an archetype of lung milieu and ailing prognosticator of ARDS^19,20,28–31^. Therefore, in our study we used both the biological matrices as diagnostic fluid in the identification disease specific metabolic endotypes which can act as biological marker significant for clinical understanding and distinguishing ARDS heterogeneity. The heterogeneity and pathogenesis hampers the tailored and directed therapies. Therefore, to make a clear interpretation and distinction responsible of same phenotypic lung injury corresponding to different aetiology and pathophysiology depends on targeting biological process or metabolic pathway for better-targeted therapies and clinical management. Most of the recruitment manoeuvres are focused on the cure but the goal should be focussed towards finding cause due to marked physiological difference between ARDS subtypes. Therefore, large sample size has been incorporated in the present study with validation in training set and test set so that results can be generalized for widespread population for clinical utility.

Following the analysis, the metabolites common to markers of severity in subphenotype1 and sensitive to etiological causes of direct and indirect ARDS in subphenotype2 were grouped to determine their analogy in predicting mortality in survivor and non-survivor group. Their discriminating power in both biofluids were checked to determine the dysregulated pathobiological mechanism occurring as a consequence of ARDS. The final panel of metabolites obtained, representing the mBALF and serum endotypes reflected the perturbed biological mechanism corresponding to subphenotype. Their clinical utility and accuracy was further validated by combing with APACHE and SOFA score in governing the mortality score. Subsequently the pathway analysis in mBALF and serum resulted in determining the affected biological mechanism which was integrated to common biological pathway to aid its correlation to outcome and clinical predictability. The distinct set of metabolic endotypes grouped together as mBALF and serum endotype were found to have important role in ARDS pathogenesis as observed in its overrepresentation in pathway analysis.

The class of serum endotypes included proline, glutamate, phenylalanine and valine. Their putative role has also been observed and recognized in previous corresponding ARDS studies. Proline upregulation in the form of proline-glycine-proline have been found to be interlinked to ARDS^32^. Proline has been established as a substratum of stress and thus owing to subsequent inflammation, it is used as energy source^33^. Though glutamate is found in lesser concentration in plasma, it governs signal mediation in peripheral organs and tissues. Glutamate metabolism has been linked to immunomodulation with its prominent role as glutamate receptors in mediating T-cell mediated immunity. In a recent study by Evans *et al*.^34^ and Rogers *et al*.^35^, both proline and glutamate have been associated with increased abnormality and correlated to dysfunction metabolism in ARDS. Increased concentration of phenylalanine has been determined both by NMR and GC-MS in distressed lung of asthmatic diagnosed patients^21,36^. Phenylalanine levels were also found associated in ARDS caused by H1N1 pneumonia by virtue of resultant viral and bacterial infections^37^. Protein breakdown is widely associated in sepsis and trauma which are among the major etiologies associated with ARDS in which protein involved in alveolar epithelium and surfactant dysfunction has been widely researched. Surfactant protein C is one of the widely studied marker which is mainly composed of valine, leucine and isoleucine forming the bulk of its hydrophobic residues^38^. Disproportion of amino acid in plasma is widely manifested by critically ill patients with deficit of BCA reported to unbalance the homeostasis of transferrin, nitrogen retention and lymphocyte counts. Overrepresented metabolic variation of these serum endotype is predominately influencing ARDS biochemical process through nitrogen metabolism, aminoacyl tRNA synthesis, arginine and proline metabolism, valine leucine and isoleucine synthesis, pantothenate and CoA synthesis, D-Glutamine and D-glutamate metabolism, phenylalanine, tyrosine and tryptophan biosynthesis.

The class of mBALF endotypes included lysine, arginine, tyrosine, threonine and branched chain amino acids (BCA). These individual metabolic endotypes have been previously reported in studies related to ARDS and its etiologies emphasizing their notable role in ARDS biology. Surge in the percentage of lysine and arginine has been observed in bronchoalveolar lavage fluid corresponding to dysfunction of diamino transport affecting the alveolar epithelial lining fluid and alveolar epithelial cells. This particular mutation has been proposed to govern the increase in the prevalence of ARDS^39^. Arginine and its interplay with nitric oxide (NO) pathway has also been found to have important implication and its corresponding regulation of multiple organ dysfunction syndrome in sepsis associated ARDS^40^. When considering the pathogenesis and exacerbated inflammation in ARDS, NO plays a vital role as oxidant stress whose metabolic by-products proves to be an important marker in early stage of ARDS. Accordingly, tyrosine upregulation in ARDS has been correlated to portending ARDS incidence prior to disease development for its paramount role as NO_2_Tyr in BAL^41^. Also, many growth factors preferably utilize serine threonine and tyrosine kinase receptors for repair and resolution of ARDS by recruiting tyrosine and threonine in the signalling pathway^42^. BCA includes isoleucine, leucine and valine which are vital components of immune enhancing formula with imperative role in muscle rejuvenation, nitrogen balance and protein synthesis^43^. Energy deficit associated with sepsis and tissue injury gravitates increased BCA oxidation by skeletal muscle. Hypermetabolic composition of these mBALF endotype mirrors anomalous metabolism in ARDS reflected prominently in nitrogen metabolism, aminoacyl tRNA synthesis, valine leucine and isoleucine synthesis and degradation, pantothenate and CoA synthesis, tyrosine metabolism, phenylalanine, tyrosine and tryptophan biosynthesis.

This study is one of the first attempts with an all-inclusive metabolic profile of different ARDS subtype in different biofluid. mBALF represents the biomatrix that can best represent the clinical phenotype of the distressed lung due to its close proximity to alveoli and its associated anomaly. Serum is the circulating biofluid phenotyping the immediate perturbations outspreading to from the diffused lung due to infection, inflammation and multiple pathogenesis. Thus both biofluids were used to fingerprint ARDS heterogeneity. Clinical phenotype was checked and validated in large population with its robustness cross validated by statistical analysis. The endotype determined from both subtypes were probed with respect to outcome in survivor and non-survivor subjects and it correlation to clinical based mortality score was well substantiated. Resultant mBALF based endotype model included lysine, arginine, tyrosine, threonine and branched chain amino acids and serum endotype model included proline, glutamate, phenylalanine and valine. Consequently, serum and mBALF endotypes illustrated perturbation mainly in TCA cycle intermediates, protein synthesis and its breakdown products and the corresponding nitrogenous network mirroring the complex biological process involved in ARDS through pathway analysis and its interconnected metabolism. Thus this study is the first of its kind to depict the complete metabolic endotype of ARDS heterogeneous phenotype with its correlation to mortality.

In conclusion, biological insight is imperative to understand heterogeneity for ARDS for early prediction, prevention and progression of ARDS to severity. Physiology driven endotypes are the answer to provide biomarkers which can discriminate ARDS subsets of patients at risk and target therapy accordingly. Characteristic endotypes of ARDS phenotypes will aid in enrolment of patients on the basis of stratified risk, follow up studies and future clinical trials. Thus in this study a model based on metabolic endotype (mBALF and serum endotype) was probed which holds potent prospects in addressing the underlying heterogeneity in ARDS subtypes. This study provides a comprehensive metabolic endotypes (lysine, arginine, tyrosine, threonine and branched chain amino acids proline, glutamate, phenylalanine and valine) associated with ARDS subtype, complementing clinical score and significant predictability in outcome. It can be separately validated in different ethnic groups for its validation and applicability for further bench to bedside prospects.

## Materials and Methods

### Ethical approval

The study was designed as a part of prospective single centre cohort performed at Intensive Care Unit (ICU) of a tertiary care medical centre, Sanjay Gandhi Post Graduate Institute of Medical Sciences (SGPGIMS), Lucknow, and Centre of Biomedical Research (CBMR), Lucknow, India. The SGPGIMS ethical body reviewed and approved the experimental methodology. A prior written informed consent was obtained from all subjects or their surrogate decision makers before the sample procurement. We confirm that all methods were performed in accordance with the relevant guidelines and regulations approved by the ethical committee of SGPGIMS.

### Diagnostic criteria

All the diagnosis was carried out by expertise and trained resident doctors at ICU unit following the standard guidelines aforementioned in the Berlin definition^44^. Lung protective ventilation measures were practiced in the ICU. Exclusion criteria in the current study include patients with age less than 18 years, pregnancy, chronic obstructive pulmonary disease (COPD) patients, bronchial asthma, interstitial lung disease, and other chronic respiratory ailments.

### Subjects and clinical design

Total n = 464 samples including n = 199 mBALF and n = 265 serum samples were collected in the study during the period from 2014–2017 from both control patient and patient diagnosed with ARDS enrolled at ICU admission of SGPGIMS. The control group comprised patients who were on mechanical lung ventilation for routine elective surgeries. Both the biofluid were collected to provide a complete overview of inflammatory markers of lung origin as well as serological markers spread through bloodstream. mBALF is a non-directed lung lavage collected from proximal alveoli which best represents the pulmonary markers of lung compartment. Standardized “catheter in catheter” technique was employed in ICU by trained medical practitioner for collection of mBALF sample from mechanically ventilated patients within 24 hours of diagnosis^45^. The mBALF sampling were performed in accordance with the approved guidelines and regulations of the institute’s ethical committee. The procedure was followed under safety guidelines using soft suction, nontoxic, pyrogen free, graduated 16 French gauge/8 French gauge catheter in catheter set. Sterile aseptic precautions were followed for mBALF suctioning using 10 ml sterile distilled water in mucus trap which is then transferred to collection vial and quenched in liquid nitrogen till further processing. A total of 2.0 ml blood was collected for each sample taken from ARDS diagnosed patients and control patients in the storage vial till further NMR analysis were performed.

### Sample preparation

Before NMR analysis, all the mBALF samples were vortexed and then centrifuged at 16000 rpm for 10 min at 4°C to remove cellular debris and bacteria. The supernatant was pipetted out and cryoprotected at −80 °C inside freezer till further NMR experiments were performed. In case of serum, all samples were incubated for 30 min at room temperature and then centrifuged at 6708 * g; 4 °C for 5 min. Golden yellow supernatant is collected and preserved at −80 °C till further NMR experiments. All the processed samples aforementioned before were suitably thawed before NMR data acquisition.

### NMR spectroscopy of mBALF and serum

All the experiments were recorded at 800 MHz NMR spectrometer having cryogenically cooled triple-resonance TCI (^1^H, ^13^C, ^15^N, and ^2^H lock) probe maintained at 300.15 K. For mBALF NMR analysis, a total of 550 µl sample was taken including 350 µl mBALF sample and 200 µl buffer (0.1 M Na_2_HPO_4_/NaH_2_PO_4_, pH-7.4) containing 6.53 mM Trimethylsilylpropanoic acid (TSP), D_2_O. One dimensional water presaturation pulse sequence noesygppr was used for mBALF experiments with acquisition parameters of 128 scans, 20 ppm spectral width and 5 sec relaxation delay, 64 k data points with an acquisition time (T_aq_) of 1.99 seconds. For serum NMR experiments, a total of 450 ul serum sample with internal capillary containing 0.156 mg of TSP was prepared for external standard reference. 1D CPMG pulse sequence was used with water presaturation and optimized for excluding the broad signal from protein and lipids. The acquisition parameters included 128 transients set for 20.55 spectral width and 5 s relaxation delay collected into 64 k data points. Line broadening function of 0.3 Hz and zero filling was applied to all spectra prior to Fourier transformation.

### Assignment of mBALF and serum

The acquired 1D ^1^H spectra was referenced to TSP signal (δ = 0.00 ppm) and manually phased and baseline corrected. The characterization and assignment of peaks resonating from small molecular metabolites in ^1^H spectra of mBALF and serum were made using 2D experiments (COSY, TOCSY, HSQC) Biological magnetic resonance bank (BMRB), HMDB database and established literature values. 54 peaks in serum and 52 peaks in mBALF were consequently assigned corresponding to 68 control serum sample and 197 serum ARDS samples and 40 mBALF control samples and 159 mBALF ARDS samples.

### Metabolite profiling

Following the identification of all the possible metabolites present in diseased samples of ARDS, the multidimensional dataset generated from NMR acquisition was subjected to chemmometric analysis to deduce the candidate markers of ARDS pathobiology which otherwise couldn’t be inferred from visual inspection of NMR spectrum. Each of the NMR acquired data were subjected to normalization using log_2_ transformation and Pareto scaled before statistical analysis by Metaboanlyst^46^. Firstly, the accuracy of mBALF and serum as a diagnostic biofluid in ARDS characterization was established by univariate and multivariate analysis distinguishing control patients and diagnosed ARDS patients. After validation, diseased samples of ARDS was targeted to determine the metabolic profile or panel of putative metabolites responsible for distinguishing ARDS subtypes and predictive of mortality with important implications in manifesting aberrant biological pathway. ARDS subtypes were clinically phenotyped and grouped based on severity as mild, moderate and severe ARDS as subphenotype1 and respiratory mechanics as pulmonary and extrapulmonary ARDS as subphenotype2 in both mBALF and serum biofluid to better interpret ARDS heterogeneity. Unpaired t-test and ANOVA were used to categorize significant metabolites based on *p* value less than 0.05 in both subphenotypes and subsequently used in substantiating metabolic endotype in outcome subgroups. Multivariate PCA and PLS-DA determined VIP plot were used to establish differential metabolites with value more than 1. R2 and Q2 value was used to determine predictability and cross validation, permutation test statistics were used to validate the model. Pearson correlation based heat map were used to determine the robustness of the model with enumeration of upregulated and downregulated metabolites in subphenotypes and resultant endotype. Individual box whisker plot and the corresponding specificity and sensitivity using AUROC to determine the credentials of endotype in outcome subgroup. Clinical score was combined with model based on endotype in AUROC to validate the accuracy of the model. To determine the endotype governing biological process in ARDS, pathway analysis was carried using Metaboanalyst based on hypergeometric test using relativeness between centrality algorithms. Significant *p* value and impact value was deduced from pathway enrichment analysis and pathway topology to depict overrepresented pathways interconnected to ARDS metabolism.

## Supplementary information

Supporting Information

## Data Availability

The data set used/generated/analysed in the present study will be made available by the corresponding authors on reasonable request.
