# Supplementary material for: Distinct Metabolic Endotype Mirroring Acute Respiratory Distress Syndrome (ARDS) Subphenotype and its Heterogeneous Biology
Source: Sci Rep. 2019 Feb 14;9:2108. doi: 10.1038/s41598-019-39017-4 (PMC6375936; doi:10.1038/s41598-019-39017-4)
Supplement: Supplementary file 1 — Supporting Information [file 41598_2019_39017_MOESM1_ESM.pdf]

Supporting Informations

Distinct Metabolic Endotype Mirroring Acute Respiratory Distress Syndrome (ARDS) Subphenotype and its Heterogeneous Biology

Akhila Viswan[1,2], Pralay Ghosh[3], Devendra Gupta[4], Afzal Azim[3*] and Neeraj Sinha[1*]*

[1]Centre of Biomedical Research
SGPGIMS Campus, Raebarelly Road
Lucknow – 226014 INDIA

[2]Faculty of Engineering and Technology, Dr. A. P. J. Abdul Kalam Technical University, Lucknow 226021, INDIA

[3]Department of Critical Care Medicine
Sanjay Gandhi Postgraduate Institute of Medical Sciences
Lucknow – 226014 INDIA

[4]Department of Anaesthesia
Sanjay Gandhi Postgraduate Institute of Medical Sciences
Lucknow – 226014 INDIA

*Author to whom correspondence should be addressed;
Dr. Devendra Gupta (dgupta@sgpgi.ac.in)
Dr. Afzal Azim (draazim2002@gmail.com , afzala@sgpgi.ac.in)
Dr. Neeraj Sinha (neerajcbmr@gmail.com , neeraj.sinha@cbmr.res.in)

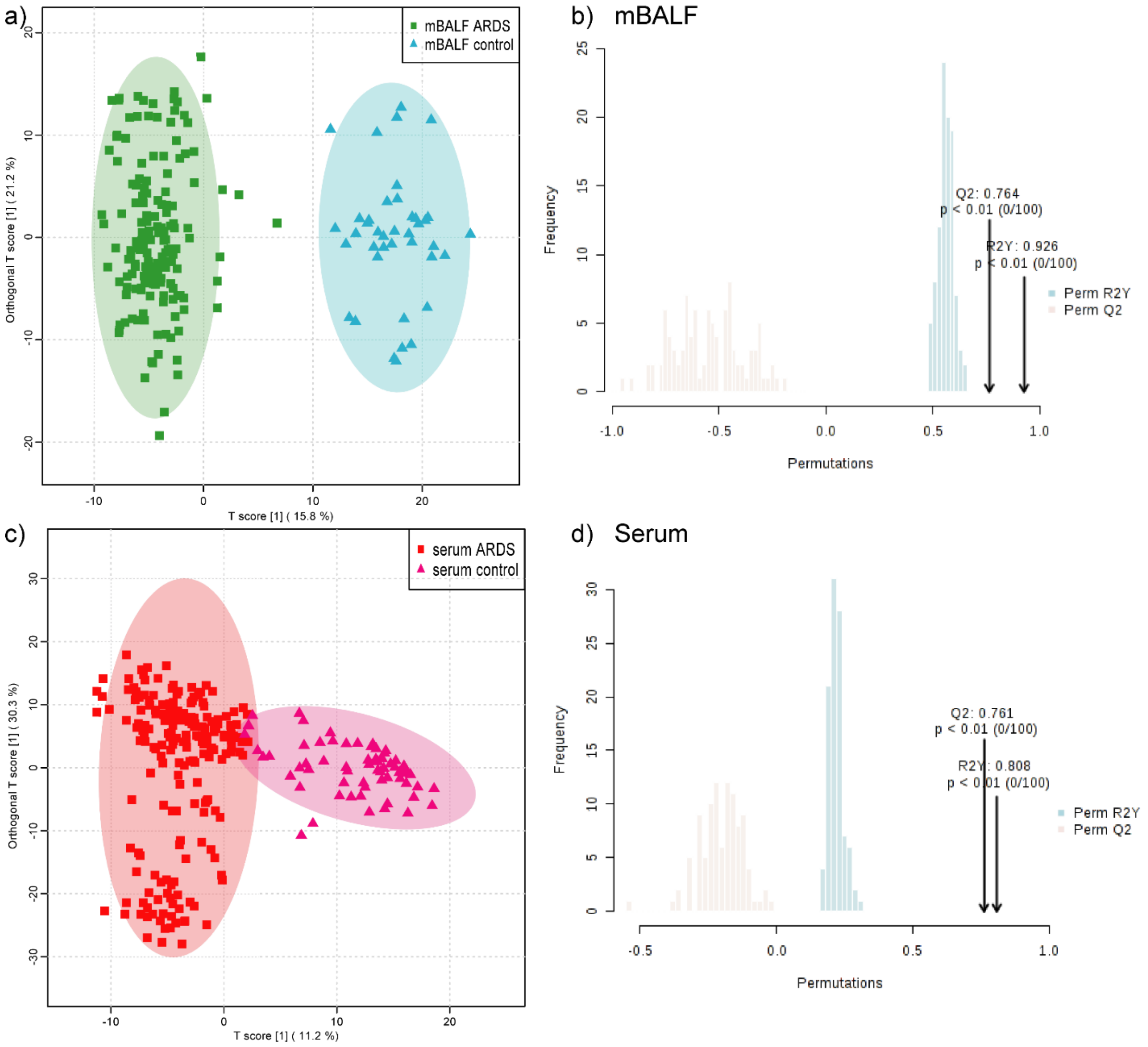

SI Figure 1: OPLS-DA based separation of ARDS patients with respect to control in **a and b)** mBALF and **c and d)** serum.

a)

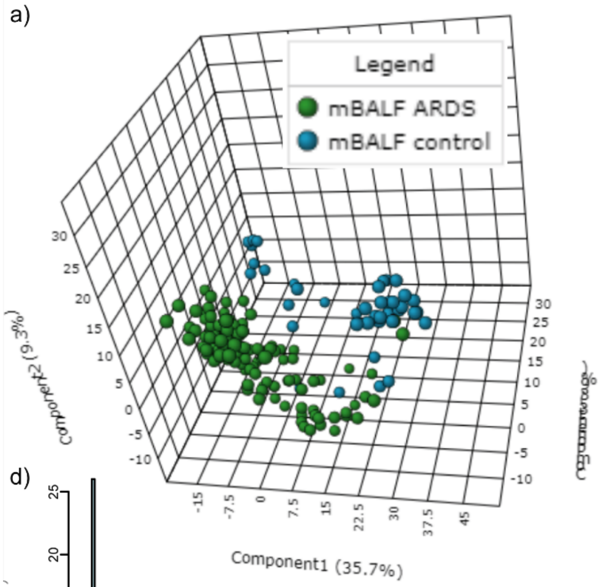

b)

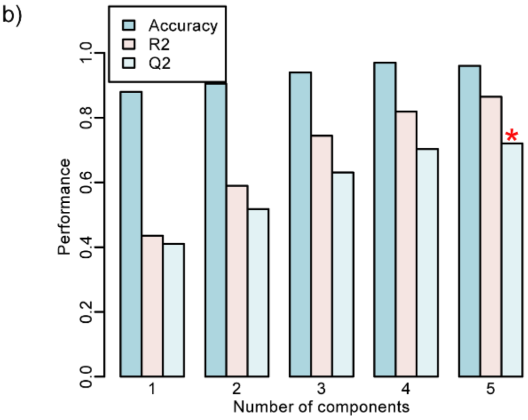

d)

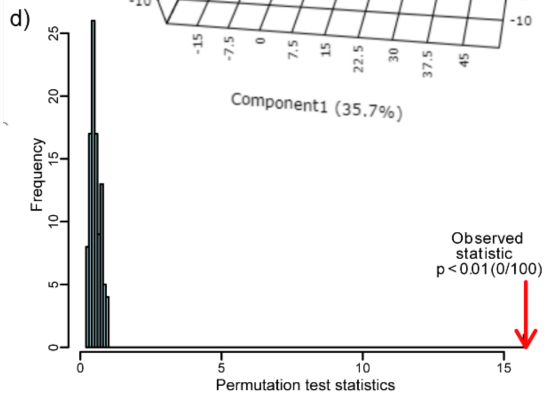

c)

PLS-DA cross validation details:

Measure	1 comps	2 comps	3 comps	4 comps	5 comps
Accuracy	0.8794	0.90452	0.9397	0.96985	0.9598
R2	0.43515	0.58928	0.74377	0.81847	0.86452
Q2	0.4099	0.51773	0.6308	0.70316	0.71995

e)

The OOB error is 0.0754

	mBALF ARDS	mBALF control	class.error
mBALF ARDS	154	5	0.0314
mBALF control	10	30	0.25

SI Figure2: PLS-DA based separation of ARDS patients with respect to control using mBALF a) 3D score plot, b) and c) cross validation accuracy, R2 and Q2 values using leave one out cross validation (LOOCV) with asterisk denoting the best classifier d) permutation test statistics using separation distance based on sum of squares between and sum of squares within (B/W) ratio and e) classification error rate.

a)

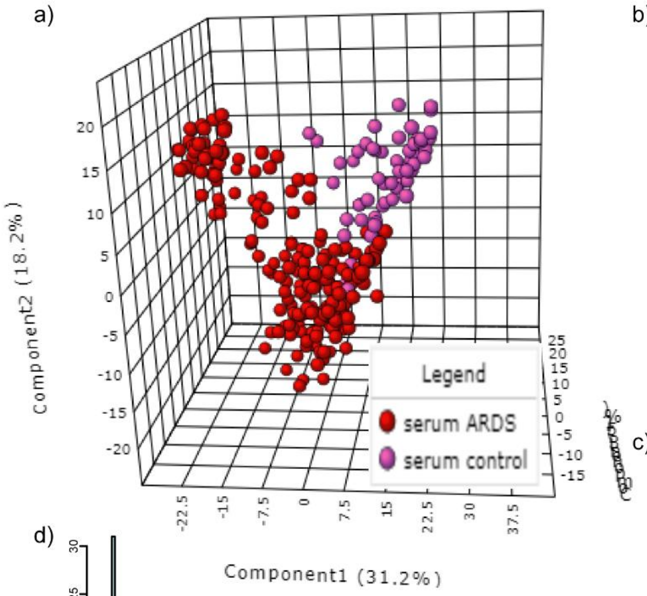

b)

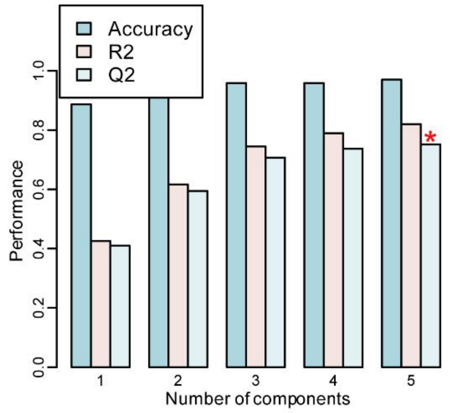

c)

PLS-DA cross validation details:

Measure	1 comps	2 comps	3 comps	4 comps	5 comps
Accuracy	0.88679	0.90943	0.95849	0.95849	0.96981
R2	0.42569	0.61626	0.74463	0.78937	0.8195
Q2	0.4097	0.59445	0.70683	0.73678	0.75164

d)

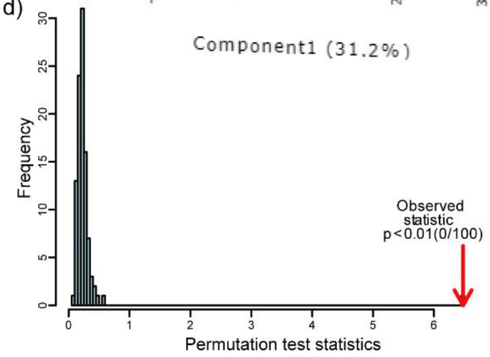

e)

The OOB error is 0.0604

	serum ARDS	serum control	class.error
serum ARDS	197	0	0.0
serum control	16	52	0.235

SI Figure 3: PLS-DA based separation of ARDS patients with respect to control using serum a) 3D score plot, b) and c) cross validation accuracy, R2 and Q2 values using leave one out cross validation (LOOCV) with asterisk denoting the best classifier d) permutation test statistics using separation distance based on sum of squares between and sum of squares within (B/W) ratio and e) classification error rate.

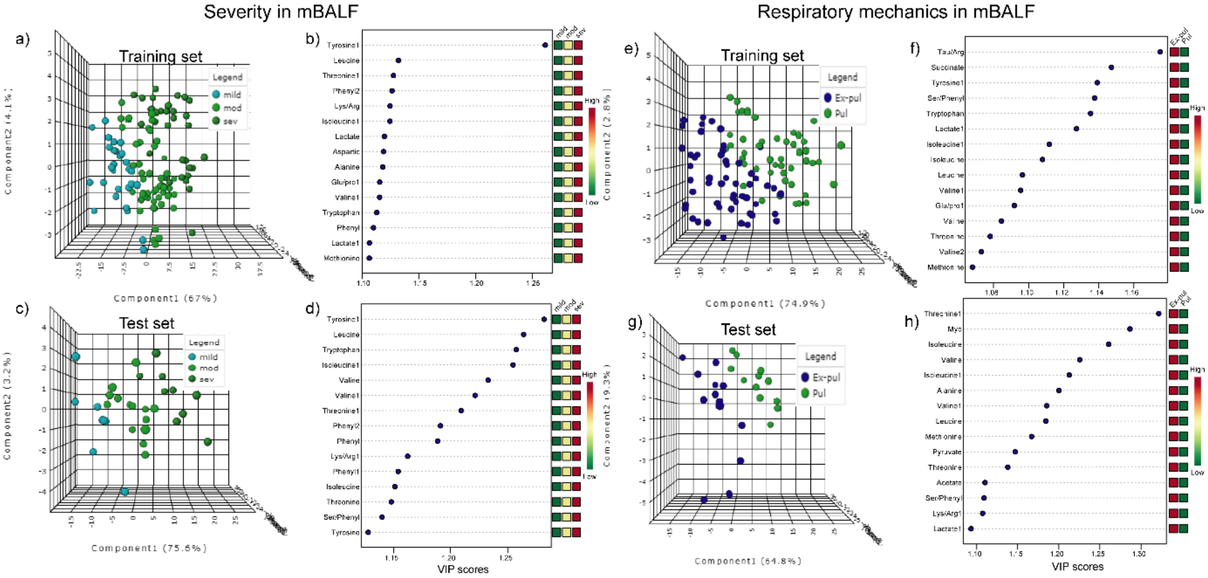

SI Figure 4: Training set and test in subphenotype1 and subphenotype 2 of mBALF

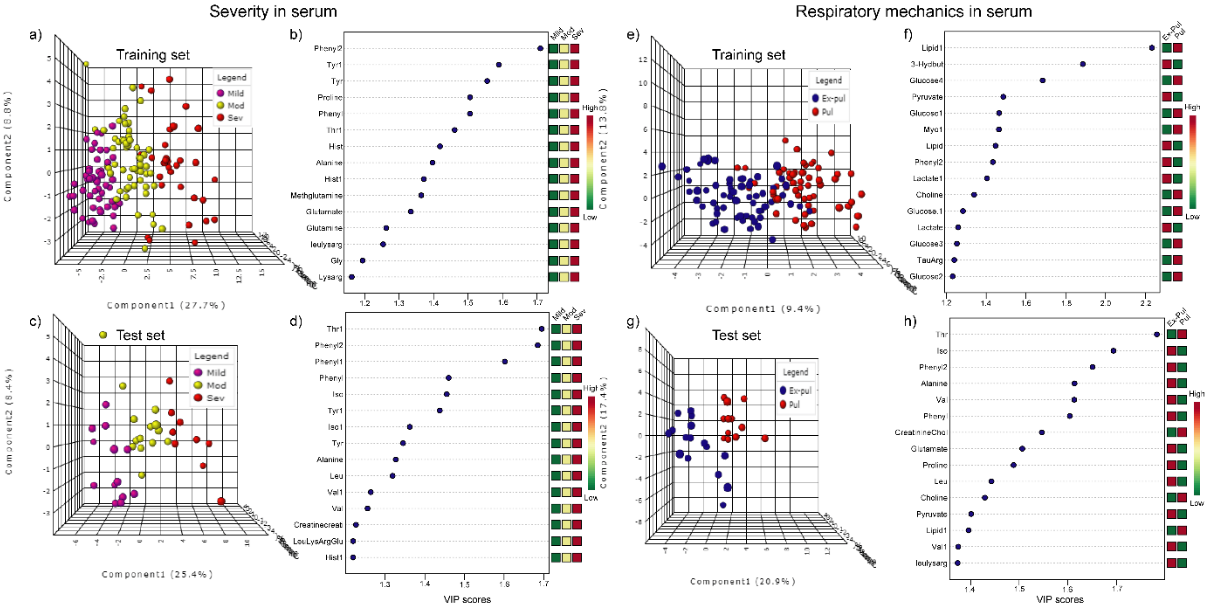

SI Figure 5: Training set and test in subphenotype1 and subphenotype 2 of serum

Subphenotype 1

a)

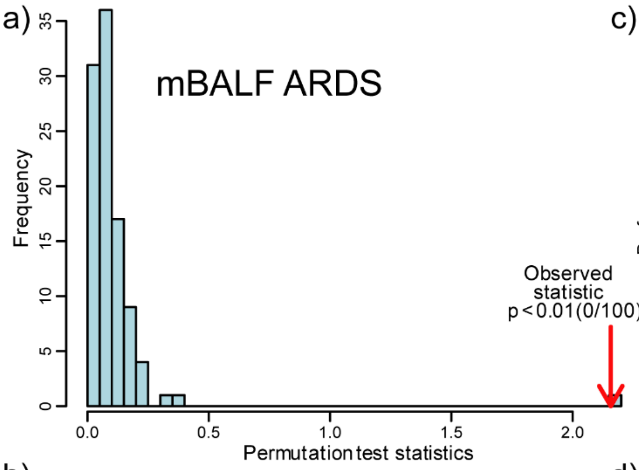

c)

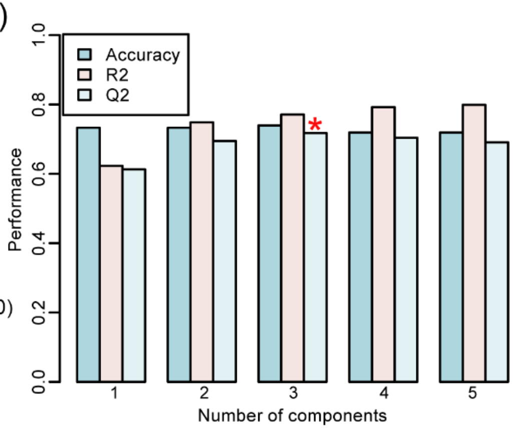

b) The OOB error is 0.219

	mild	mod	sev	class.error
mild	32	4	0.0	0.111
mod	6	51	9.0	0.227
sev	0	13	31.0	0.295

d) **PLS-DA cross validation details:**

Measure	1 comps	2 comps	3 comps	4 comps	5 comps
Accuracy	0.73288	0.73288	0.73973	0.71918	0.71918
R2	0.62312	0.7484	0.77087	0.79225	0.7991
Q2	0.6127	0.6946	0.71756	0.70414	0.69077

e)

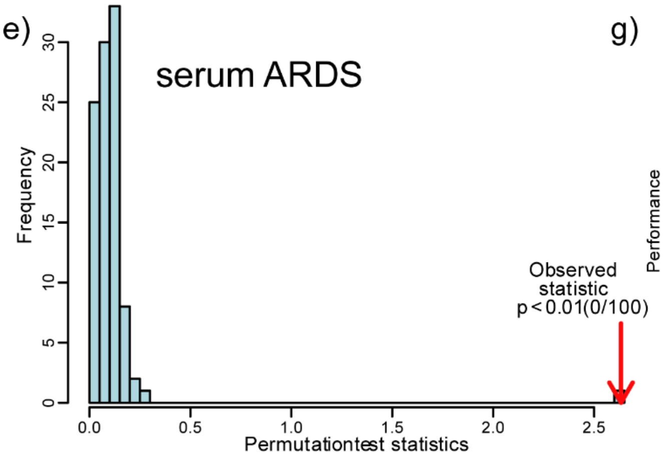

g)

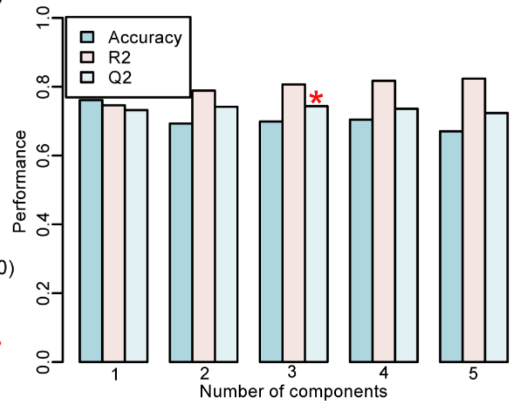

f) The OOB error is 0.199

	Mild	Mod	Sev	class.error
Mild	49	13	0.0	0.21
Mod	11	57	4.0	0.208
Sev	0	7	35.0	0.167

h) **PLS-DA cross validation details:**

Measure	1 comps	2 comps	3 comps	4 comps	5 comps
Accuracy	0.76136	0.69318	0.69886	0.70455	0.67045
R2	0.74603	0.78859	0.80684	0.81728	0.82351
Q2	0.73195	0.74189	0.74357	0.73585	0.72368

SI Figure 6: PLS-DA based classification of ARDS Subphenotype 1 using mBALF and serum a) permutation test statistics using separation distance based on sum of squares between and sum of squares within (B/W) ratio b) classification error rate c) and d) cross validation accuracy, R2 and Q2 values using leave one out cross validation (LOOCV) with asterisk denoting the best classifier

Subphenotype 2

a)

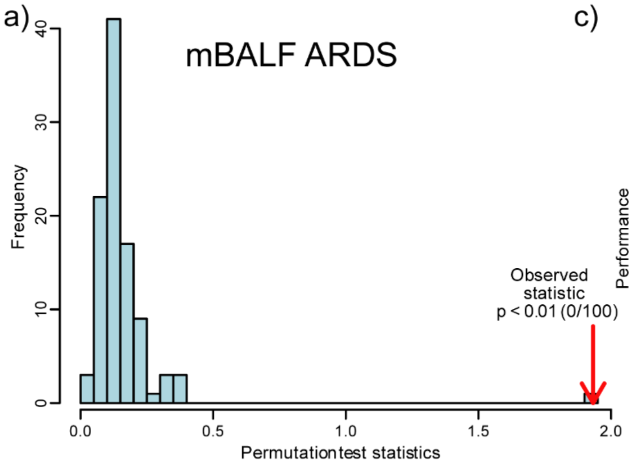

c)

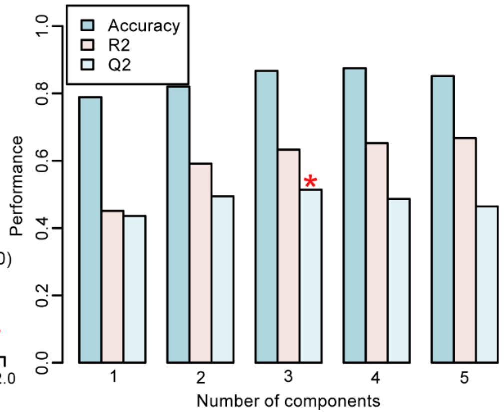

b)

The OOB error is 0.156

	Ex-pul	Pul	class.error
Ex-pul	61	7	0.103
Pul	13	47	0.217

d) **PLS-DA cross validation details:**

Measure	1 comps	2 comps	3 comps	4 comps	5 comps
Accuracy	0.78906	0.82031	0.86719	0.875	0.85156
R2	0.451	0.59156	0.63279	0.65218	0.66735
Q2	0.43617	0.49448	0.51383	0.48649	0.46434

e)

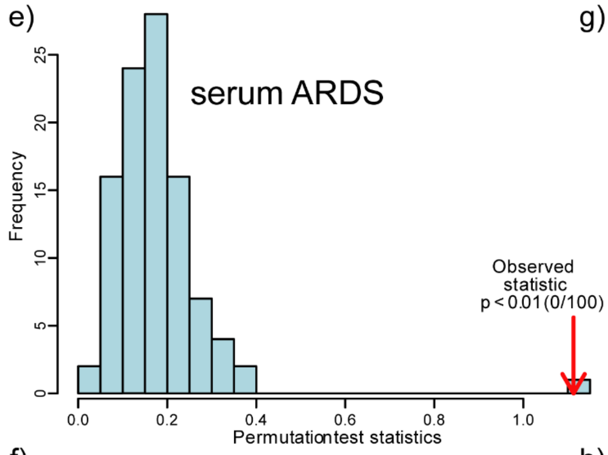

g)

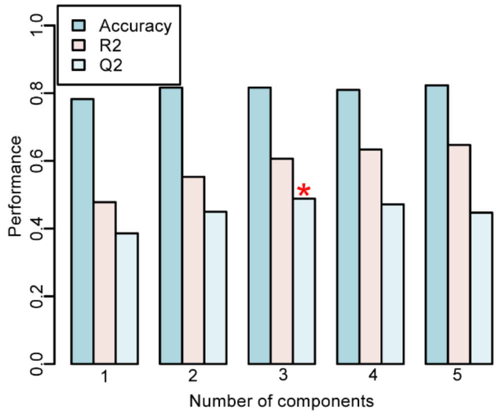

f)

The OOB error is 0.231

	Ex-Pul	Pul	class.error
Ex-Pul	65	15	0.188
Pul	19	48	0.284

h) **PLS-DA cross validation details:**

Measure	1 comps	2 comps	3 comps	4 comps	5 comps
Accuracy	0.78231	0.81633	0.81633	0.80952	0.82313
R2	0.47803	0.55285	0.60622	0.63347	0.64688
Q2	0.38611	0.44943	0.48814	0.47168	0.4469

SI Figure 7: PLS-DA based classification of ARDS Subphenotype 2 using mBALF and serum a) permutation test statistics using separation distance based on sum of squares between and sum of squares within (B/W) ratio b) classification error rate c) and d) cross validation accuracy, R2 and Q2 values using leave one out cross validation (LOOCV) with asterisk denoting the best classifier

ARDS endotype accuracy

a)

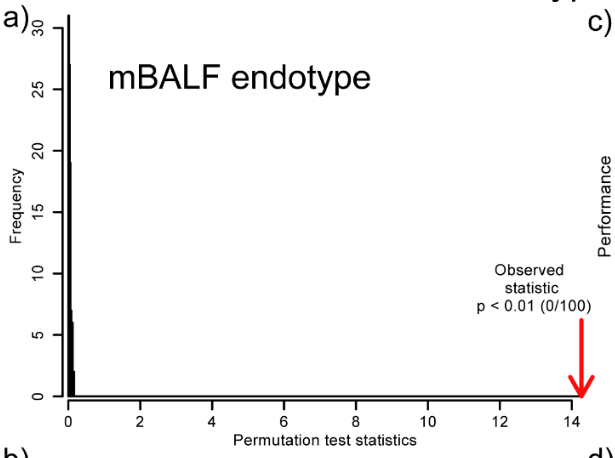

c)

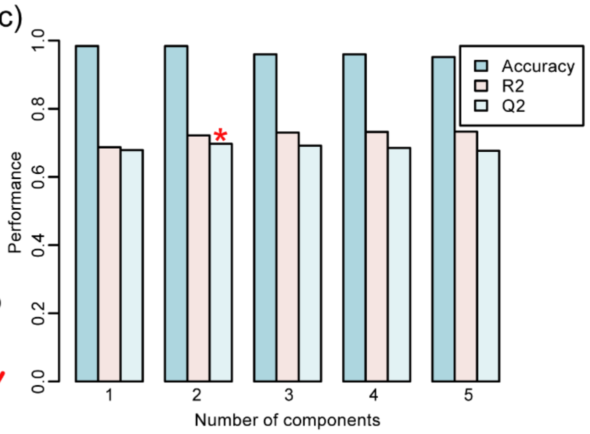

b)

The OOB error is 0.0242

	NS	S	class.error
NS	72	1	0.0137
S	2	49	0.0392

d) **PLS-DA cross validation details:**

Measure	1 comps	2 comps	3 comps	4 comps	5 comps
Accuracy	0.98387	0.98387	0.95968	0.95968	0.95161
R2	0.68736	0.72177	0.73002	0.73192	0.73289
Q2	0.67866	0.69701	0.69178	0.6852	0.67666

e)

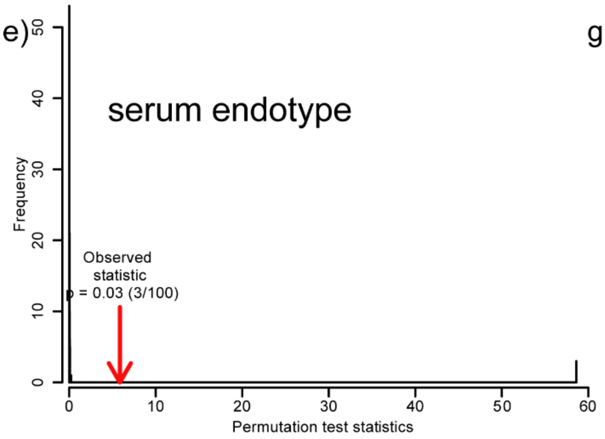

g)

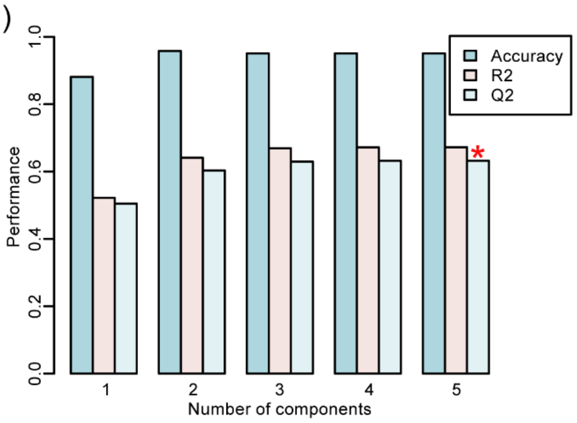

f)

The OOB error is 0.049

	NS	S	class.error
NS	89	3	0.0326
S	4	47	0.0784

h) **PLS-DA cross validation details:**

Measure	1 comps	2 comps	3 comps	4 comps	5 comps
Accuracy	0.88112	0.95804	0.95105	0.95105	0.95105
R2	0.52181	0.64101	0.6692	0.67182	0.67233
Q2	0.50469	0.60309	0.62932	0.63191	0.63199

SI Figure 8: PLS-DA based classification of ARDS endotype accuracy in outcome group using mBALF and serum a) permutation test statistics using separation distance based on sum of squares between and sum of squares within (B/W) ratio b) classification error rate c) and d) cross validation accuracy, R2 and Q2 values using leave one out cross validation (LOOCV) with asterisk denoting the best classifier

SI Table 1: List of significant metabolites from subphenotype 1 based on p-value less than 0.05 and VIP value more than 1 mBALF with mean values and standard deviation (before normalization)

**mBALF Metabolites**						
Subphenotype1	***mild***		***moderate***		***severe***	
	mean	*Standard deviation*	*mean*	*Standard deviation*	*mean*	*Standard deviation*
Lysine/arginine	0.012169	0.007415	0.078341	0.066973	0.34336	0.3138
Alanine	0.005156	0.005228	0.037955	0.037618	0.178903	0.176955
Isoleucine	0.001164	0.00087	0.007964	0.007417	0.03555	0.036611
Leucine	0.004061	0.003318	0.043275	0.043118	0.238137	0.262672
Phenylalanine	0.001071	0.000921	0.008873	0.007744	0.042832	0.047754
Valine	0.002031	0.001499	0.019289	0.01846	0.097532	0.106119
Tyrosine	0.000629	0.000439	0.00659	0.006568	0.034283	0.036911
Glutamate/proline	0.003369	0.003407	0.020823	0.017822	0.095154	0.090455
Aspartic	0.011222	0.013375	0.041116	0.035522	0.156485	0.162671
Tryptophan	0.000394	0.000332	0.001687	0.001396	0.006037	0.004775
Threonine	0.000971	0.001092	0.005734	0.005411	0.033394	0.042501
Serine/Phenyalnanine	0.004166	0.004692	0.025473	0.020549	0.105169	0.104072
Lactate	0.019944	0.028442	0.087738	0.071994	0.433998	0.464182
Methionine	0.001091	0.001247	0.004809	0.004371	0.025617	0.025626

SI Table 2: List of significant metabolites from subphenotype 2 based on p-value less than 0.05 and VIP value more than 1 in mBALF with mean values and standard deviation (before normalization)

mBALF Metabolites				
Subphenotype2	***Pulmonary***		***Extra-pulmonary***	
	mean	*Standard deviation*	*mean*	*Standard deviation*
Isoleucine	0.003984	0.005231	0.032895	0.043348
Leucine	0.01758	0.021587	0.143132	0.182675
Valine	0.006829	0.008362	0.050821	0.062326
Lactate	0.199706	0.463038	0.976946	1.024415
Lysine/Arginine	0.003055	0.003872	0.021014	0.033476
Methionine	0.002231	0.002727	0.017419	0.022097
Pyruvate	0.002039	0.003127	0.013346	0.018534
Succinate	0.00101	0.0012	0.010017	0.013786
Betaine	0.009295	0.017794	0.028529	0.031159
Taurine/Arginine	0.026056	0.039079	0.15447	0.165946
Threonine	0.003298	0.003383	0.021351	0.022989
Serine/Phenyalanine	0.0109	0.013631	0.068718	0.070442
Myoinositol	0.005174	0.007648	0.022543	0.033901
Tyrosine	0.003072	0.003511	0.020956	0.026418
Tryptophan	0.000984	0.000934	0.003929	0.003497
Glutamate/proline	0.097994	0.113418	0.326702	0.236448

SI Table 3: List of significant metabolites from subphenotype 1 based on p-value less than 0.05 and VIP value more than 1 in serum with mean values and standard deviation (before normalization)

Serum Metabolites						
Subphenotype1	***Mild***		***Moderate***		***Severe***	
	mean	*Standard deviation*	*mean*	*Standard deviation*	*mean*	*Standard deviation*
Tyrosine	0.016352	0.005602	0.025668	0.009527	0.068086	0.04107
Phenylalanine	0.019874	0.018837	0.030272	0.014754	0.07374	0.037646
Leucine/Lysine/Arginine	0.153689	0.034298	0.236026	0.114798	0.431593	0.260736
Methglutamine	0.2025	0.076553	0.316099	0.18305	0.673881	0.428301
Alanine	0.18804	0.060092	0.31854	0.171316	0.816729	0.721617
Glutamine	0.154994	0.04871	0.236019	0.118528	0.45831	0.260013
Valine	0.110937	0.035747	0.167207	0.063029	0.259938	0.106317
Proline	0.052387	0.040119	0.080143	0.046675	0.180057	0.146465
Histidine	0.00839	0.003417	0.014564	0.009356	0.032614	0.030099
Leucine	0.11419	0.037575	0.168633	0.061464	0.285105	0.125663
Glycine	0.066863	0.032738	0.105143	0.056622	0.19544	0.137991
Glutamate	0.036876	0.030279	0.058001	0.048624	0.122331	0.105138
Threonine	0.012483	0.008406	0.022394	0.012257	0.039707	0.028581
Isoleucine	0.02605	0.010322	0.039324	0.01533	0.06009	0.029005

SI Table 4: List of significant metabolites from subphenotype 2 based on p-value less than 0.05 and VIP value more than 1 in serum with mean values and standard deviation (before normalization)

**Serum Metabolites**				
Subphenotype2	***Pulmonary***		***Extra-pulmonary***	
	mean	*Standard deviation*	*mean*	*Standard deviation*
3-hydroxybutyrate*	0.125466	0.242807	0.128531	0.068558
Lactate	2.130448	1.156023	3.208909	2.370335
Alanine	0.251564	0.102011	0.423071	0.390973
Lipid	0.441894	0.491958	0.669749	0.551153
Glutamate	0.048118	0.042541	0.070583	0.054508
Pyruvate	0.037334	0.025767	0.061304	0.041835
Choline	0.140366	0.098141	0.090691	0.070544
Glucose	0.773606	0.3508	0.621611	0.352992
Glycine	0.08501	0.042626	0.12265	0.080924
Myoinositol*	0.012098	0.011584	0.009811	0.013235
Phenylalanine	0.027446	0.021785	0.042473	0.03508
Valine*	0.153149	0.068503	0.163945	0.076532
Proline	0.068555	0.037673	0.098668	0.072025
Leucine	0.148179	0.069161	0.174753	0.073506
Isoleucine*	0.043469	0.017699	0.038744	0.017991
Threonine*	0.040995	0.024317	0.038138	0.039957

* P value more than 0.05 but found significant after normalization by log transformation and Pareto scaling
